# The 3of5 web application for complex and comprehensive pattern matching in protein sequences

**DOI:** 10.1186/1471-2105-7-144

**Published:** 2006-03-16

**Authors:** Markus Seiler, Alexander Mehrle, Annemarie Poustka, Stefan Wiemann

**Affiliations:** 1 Division of Molecular Genome Analysis, German Cancer Research Center, Im Neuenheimer Feld 580, 69120 Heidelberg, Germany

## Abstract

**Background:**

The identification of patterns in biological sequences is a key challenge in genome analysis and in proteomics. Frequently such patterns are complex and highly variable, especially in protein sequences. They are frequently described using terms of regular expressions (RegEx) because of the user-friendly terminology. Limitations arise for queries with the increasing complexity of patterns and are accompanied by requirements for enhanced capabilities. This is especially true for patterns containing ambiguous characters and positions and/or length ambiguities.

**Results:**

We have implemented the 3of5 web application in order to enable complex pattern matching in protein sequences. 3of5 is named after a special use of its main feature, the novel n-of-m pattern type. This feature allows for an extensive specification of variable patterns where the individual elements may vary in their position, order, and content within a defined stretch of sequence. The number of distinct elements can be constrained by operators, and individual characters may be excluded. The n-of-m pattern type can be combined with common regular expression terms and thus also allows for a comprehensive description of complex patterns. 3of5 increases the fidelity of pattern matching and finds ALL possible solutions in protein sequences in cases of length-ambiguous patterns instead of simply reporting the longest or shortest hits. Grouping and combined search for patterns provides a hierarchical arrangement of larger patterns sets. The algorithm is implemented as internet application and freely accessible. The application is available at .

**Conclusion:**

The 3of5 application offers an extended vocabulary for the definition of search patterns and thus allows the user to comprehensively specify and identify peptide patterns with variable elements. The n-of-m pattern type offers an improved accuracy for pattern matching in combination with the ability to find all solutions, without compromising the user friendliness of regular expression terms.

## Background

The availability of complete genome sequences from several organisms [1-5] and complementing transcriptomes [6-10] has facilitated the identification of genes and of highly complex biological patterns such as novel domain regions and binding or localization motifs. Several web-based tools are available that allow for pattern matching in query sequences. Smart [11], Prosite [12], CDD [13], and Pfam [14] contain libraries of predefined patterns. Frequently, these patterns are formulated as Hidden Markov Models (HMMs) [15]. Other applications like Prosite [12], Kangaroo [16], PatSearch [17], PepPat [18] and PatMatch [19] allow users to define their own patterns to be searched for via regular expression (RegEx)-like terms [20].

The complexity of patterns within protein sequences is a major problem in pattern matching when a mixture of rigid and variable information occurs in pattern descriptions. In most applications complex patterns are handled by simplifying the expression of these patterns. This is especially the case, where the arrangement of positions and content are variable and would thus allow for an extended set of solutions. However, this simplification frequently results in a loss of information and some pattern specifications are even disregarded in pattern matching. A comprehensive formulation also of complex pattern elements would consequently minimize the number of false matches. A second problem occurs in case of length-ambiguous patterns. Commonly only the longest or the shortest hit is identified in cases where more than one match starts at the same position in a protein sequence. Analysis requires to be done in two separate processes to obtain these hits while any hit of intermediate length is not detected. An enhanced fidelity would thus be desirable especially in cases of length-ambiguous patterns. Finally, the formulation of more sophisticated patterns should be simple enough to meet the requirements especially of users lacking deep knowledge about algorithms.

Existing web-based applications miss at least one of these aspects. On the one hand common HMM building tools [15] do not allow for the definition of both rigid and variable complex patterns in a user-friendly way. Patterns with varying arrangements of elements in position, order and content cannot be introduced without knowledge in programming. On the other hand, applications that are based on regular expressions, like Prosite, are user-friendly but do not cover the complete variability within patterns. The construction of special algorithms is an alternative route but applications of these tools remain fixed to perform dedicated tasks like in Psort [21]. Finally no application is currently available via a web-based interface that would find all matches in case of length-ambiguous and user-defined peptide patterns.

Here we present the novel 3of5 web application that copes with the demands described above. It is concepted as fully on-line application to search for user-defined sets of complex peptide patterns in sets of protein sequences. For the first time, all variations of elements in a pattern stretch of user specified length can be defined in one term using the new peptide pattern type n-of-m. It permits to exclude defined amino acid characters and to set numerical constraints of distinct elements in its extended version. This is applied via two, RegEx-like expressions, one in a standard syntax, one in an extended syntax. In addition, 3of5 finds all variations in protein sequences in case of length-ambiguous patterns. Usage of 3of5 does not require theoretical background knowledge but rather enables a user-friendly and user-specified definition of terms and patterns. The algorithm is provided as interactive web-application which is freely accessible [22].

## Implementation

### Definitions

**Figure 1:**
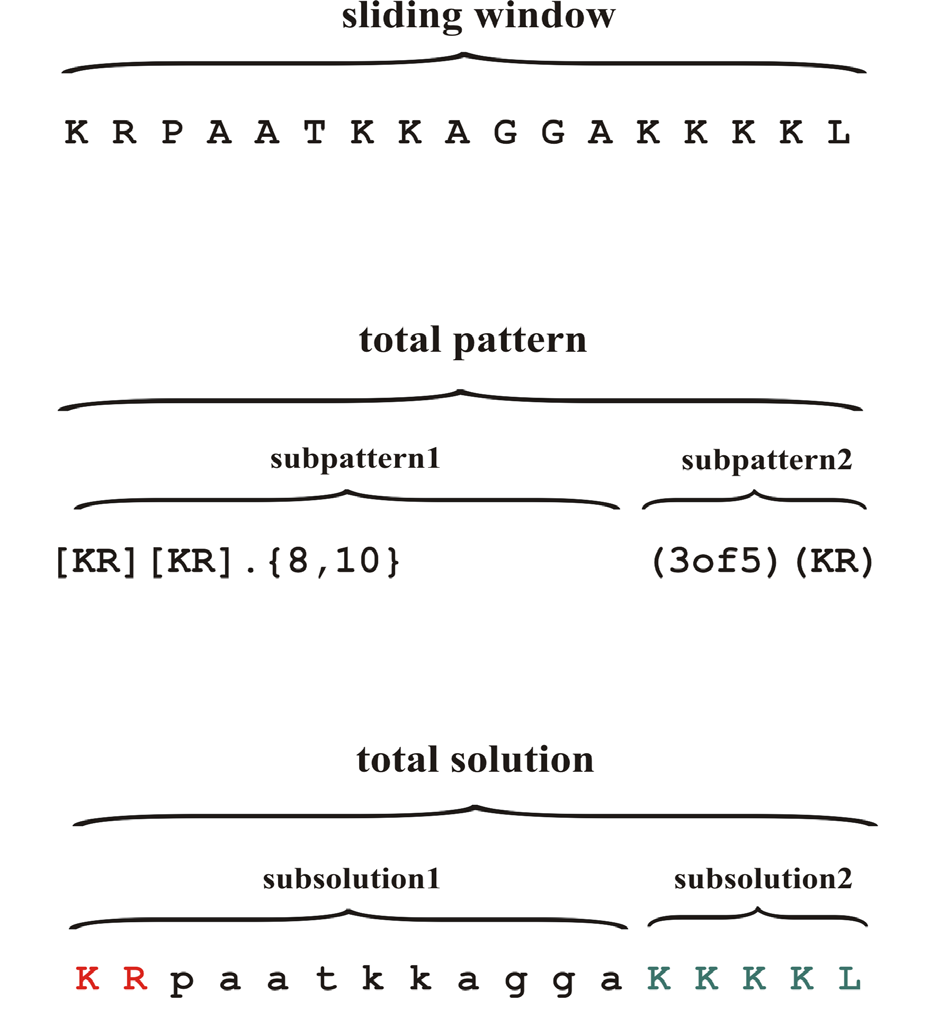
**Overview of terms. **Sequences, patterns, and solutions can be segregated to their elemental parts. The sliding window is part of the sequence that is to be searched. This size is defined by the maximal size of the total pattern. The total pattern is segregated into subpatterns that are suited for computation in the 3of5 algorithm. Matching subpatterns become a subsolution. Every branch of a solution tree becomes a total solution, once also the final subpattern has matched.

An attempt is defined as search for a pattern in a sequence. A successful attempt is called a match. Due to the modular processing of patterns and sequences, the 3of5 algorithm requires the introduction of terms on two different hierarchical levels, expressed by the denotations "sub" and "total" (Figure 1). One or more subpatterns make a total pattern. In analogy, a total solution is built of subsolutions. Length-ambiguous patterns characterize sequence stretches which vary in length. We will use the term content-ambiguous instead of "ambiguous" to emphasize the difference to length-ambiguous patterns. The attribute "length-ambiguous" is also used to describe total patterns and subpatterns which contain such length-ambiguous properties.

### The algorithm

In the preprocessing step the total pattern is initially split into its smallest parts, which may be (i) an individual character, (ii) a selected set of individual characters, (iii) the special symbol "." that can match any character, (vi) a pattern formulation of length-ambiguous sequence stretches, and (v) a pattern formulation of the n-of-m pattern type. Excluded subsets of characters are considered as part of the preceding patterns and treated as their attributes. These smallest pattern parts, once identified, are concatenated to form larger units applying the following fusion rules (1) Individual characters and content-ambiguous pattern characters are always concatenated. (2) No unit may contain more than one length-ambiguous pattern character. (3) Any n-of-m pattern forms a separate unit. Each such unit represents a "subpattern".

**Figure 2:**
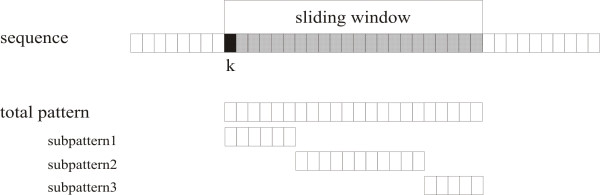
**The subpattern attempts. **The sequence of the sliding window is investigated for matches of the total pattern for every start position k individually. The total pattern is first segregated into subpatterns that are analyzed in consecutive subpattern attempts. Adjacent subpatterns may not overlap but must be consecutive. A successful subpattern attempt leads to a subsolution (not displayed), and initiates a subpattern attempt with the adjacent subpattern. A total solution is obtained when the last subpattern has led to a subsolution.

Using the sliding window mechanism every sequence position is analyzed for its potential to start a match. The actual matching processes are called subpattern attempts because they are performed consecutively at the level of subpatterns for each sliding window. A match of the first subpattern induces the corresponding subsequence to become a subsolution. Then an attempt is made to match also the second subpattern, starting at the first position of the remaining sequence, and so on. A total solution is obtained, when the last subpattern of the total pattern has matched (Figure 2). The use of subpatterns allows to process the n-of-m pattern type and to work with individual sets of subsolutions that may occur in case of length-ambiguous subpatterns. The matching process itself is performed by the RegEx terms for every subpattern. An excluded subset of characters is considered in a second step after the matching process. Exceptions of the RegEx matching process are n-of-m subpatterns, where any occurrence of a character is counted that has been specified in the pattern brackets and found in the subsequence. An n-of-m subsolution has to contain the same or higher number of matches than the defined number in the n-of-m expression in case of the standard syntax. In case of the extended syntax the type of comparative operator is user-defined.

In each subpattern attempt a length-ambiguous subpattern may generate a number of subsolutions with different end positions. Such subsolutions of the same subpattern may result in different branches of successors and distinct sets of sequences that remain to be analyzed. Such branches and branching points generate a solution tree. All consecutive subpattern attempts may have three different results: (1) A branch will be extended if also the consecutive subpattern leads to a subsolution. (2) The tree may branch again, if also a further subpattern is length-ambiguous. (3) No subsolution is found, resulting in one or more branches that terminate here. In any case, each subpattern attempt is only performed once in every sliding window.

**Figure 3:**
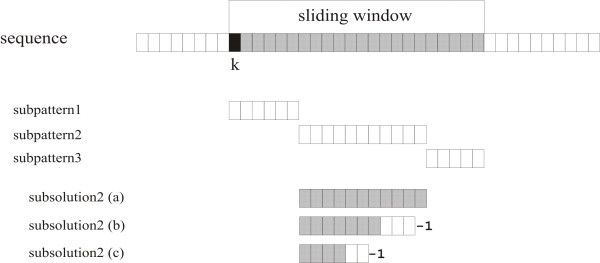
**The multivalence loop within the subpattern attempt. **Length-ambiguous subpatterns may lead to different subsolutions. A loop of subpattern attempts, the so-called multivalence loop, is initiated to iteratively find all subsolutions sharing the start position. Subpattern 2 is length-ambiguous in the schema shown. Initially the subpattern is attempted to be matched to the target sequence with its maximal size (a). Then this sequence is diminished by one position ("-1") with respect to the end of the previous subsolution (shaded stretches) to investigate, if also smaller subsolutions can be found (b, c). Note: The indicated start of subpattern 3 is only valid for subsolution 2(a). Since subsolutions are required to be directly adjacent, subsolutions 2(b) or 2(c) would require a subsolution 3 to begin immediately downstream.

In case of a length-ambiguous subpattern there is an additional cycle inside of the subpattern attempt. In this multivalence loop the decision is made about the introduction of a branch point by finding all solutions sharing the start position within the subsequence, but differing in their respective end positions. The multivalence loop begins with the longest subsolution. In successive cycles the length of the last identified subsolution is reduced by one position from the right end, and the subpattern attempt is repeated to identify any shorter subsolutions (Figure 3).

The subsolutions of the different branches are stored in a two-dimensional tree structure which contains the subpattern number and the branch number. Finally total solutions are built from the subsolutions in a backtracking step. This matching and storage process is repeated for every sliding window.

These three nested shells represent the core algorithm to match patterns in 3of5. The subpattern attempt and the multivalence loop are analogous to the sliding window principle, as these also analyze sequences within defined windows. The sliding window is always from left (N-terminus) to right (C-terminus), the individual windows are strongly overlapping and the window size is left constant. In the subpattern attempt, however, the windows are adjacent and do not overlap. The multivalence loop keeps the starting point of the window at a fixed position and successively reduces the window size from the right end in every cycle of the subpattern attempts.

### Programming environment

The 3of5 algorithm was programmed in Perl (version 5.8.5) and implemented as a CGI web-application on an Apache server (version 2.0.49) to allow easy and remote access. The Apache server is installed on a Suse Linux 9.0 server. Java scripting was implemented allowing the display of details of the input in separate windows.

## Results and discussion

### The web application 3of5

3of5 is an interactive web application that performs pattern matching in protein sequences. The user defines expressions to represent functional or structural parts of a protein sequence by using the most common subset of the Perl vocabulary of regular expressions [20]. Table 1 shows an overview of the use of these expressions. For example, the histone H2A signature [Prosite:PDOC00045] [12] is expressed as " [AC]GL.FPV". This expression combines single characters to describe discrete elements of the pattern ("G", "L", "F", "P", "V") and elements of variable yet defined content in one position (" [AC]"). The meta symbol "." allows for any character at this position. The latter two expressions are content-ambiguous, since they may have several solutions. Length-ambiguous peptide pattern elements are described as length declarations in curly brackets, like in the Succinyl-CoA Ligase pattern "G.{2}A.{4,7} [RQT] [LIVMF]GH [AS] [GH]" [Prosite:PDOC00335]. This pattern matches any sequence with an arbitrary linking segment between "A" and " [RQT]" that has a variable length of between 4 and 7 characters. A pattern of fixed length like ".{2}" is indicative of exactly two characters with arbitrary content. Further pattern features constrain the pattern matching in different manners. A subset of characters can be excluded by setting these characters in a pair of square brackets that is preceded by a "^" symbol, e.g. " [^ABC]". With 3of5 it is possible to combine this excluding subset of characters with any other content-ambiguous pattern type which is in contrast to other applications. For example, the pattern " [RQT] {4,7} [^ABC]" will match any sequence of a defined length between 4 and 7 characters that contains the characters "R","G","T", but where "A","B","C" are not allowed to occur. This option is also applicable for the n-of-m pattern type in its standard and extended versions (see below) and allows for a discrete non-matching against specific characters. In addition, the pattern matching can be constrained to the two ends of the sequence: a preceding "^" symbol limits the pattern to matches at the N-terminus of a protein sequence, a succeeding "$" symbol constrains it to the end at the C-terminus.

**Figure 4:**
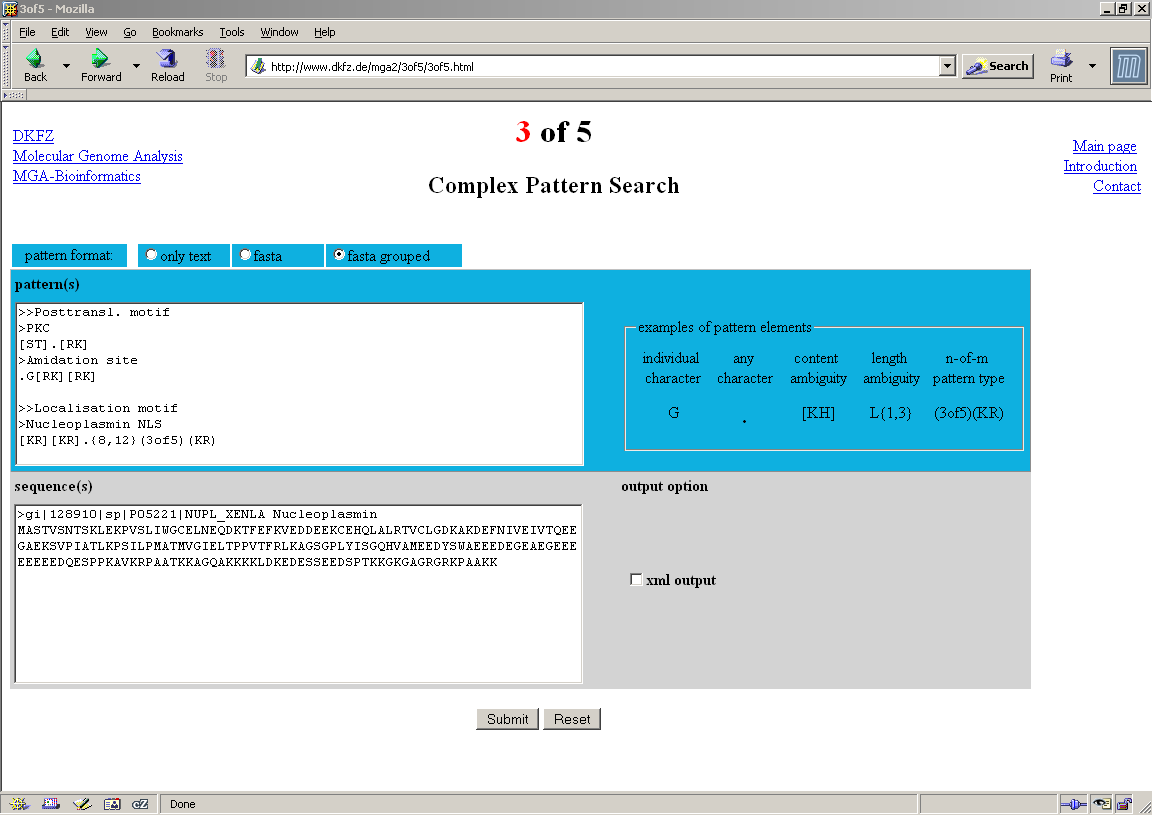
**3of5 web interface. **Three different patterns were entered to be searched for in the sequence of the nucleoplasmin protein of Xenopus laevis [Swiss-Prot:P05221]. Header lines starting with ">>" indicate grouped patterns as feature of the "FASTA grouped" mode. Two posttranslational modification patterns (PKC and Amidation) are thus combined to the group "Posttransl. motif". A second group "Localization motif" contains one pattern (nucleoplasmin NLS [Prosite:PS00015]) in the example. The pattern format is selected by activating the appropriate check box on top of the pattern window. The sequence that shall be investigated in pattern matching is copied into the sequence window, either in FASTA, multiple FASTA, or simple text formats. An output in XML is optional.

**Figure 5:**
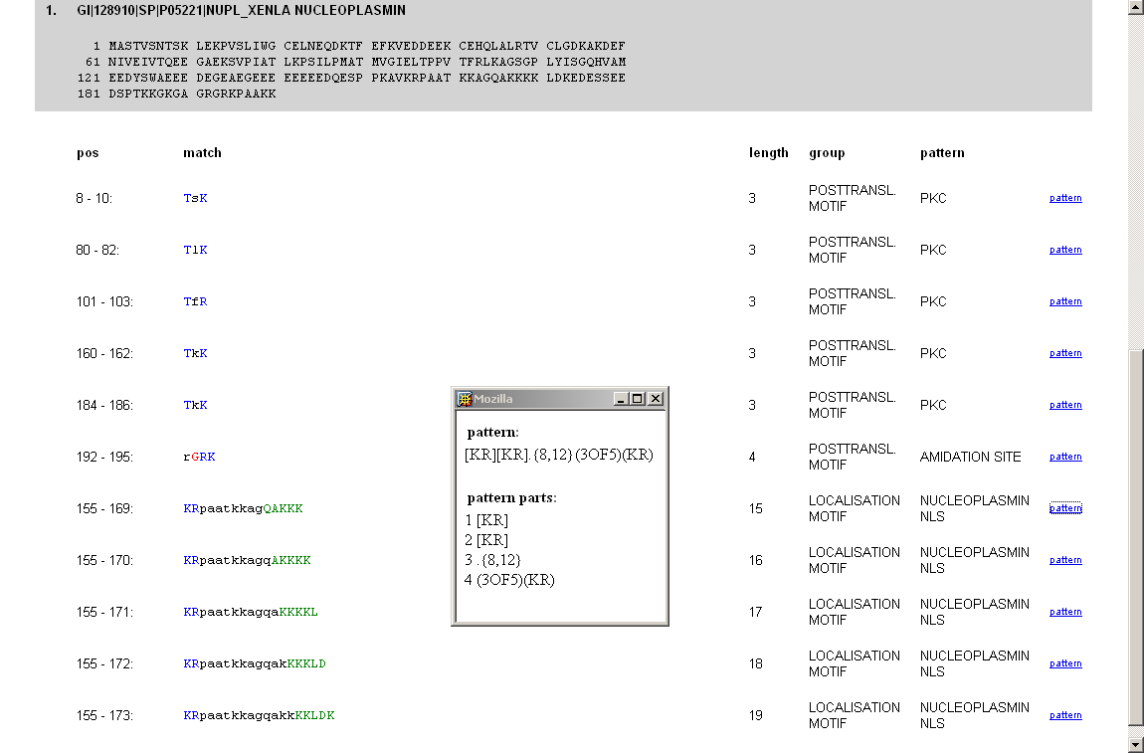
**3of5 result page for grouped patterns. **The nucleoplasmin protein of Xenopus laevis was analyzed for a set of posttranslational and localization motifs as shown in figure 1. Matches are ordered for every grouped pattern separately by their respective sequence position. A link at the right hand side opens a popup window with a detailed description of the respective pattern parts. Matches are given in a color code. Red: matching discrete characters; blue: matching characters from a subset of characters possible in one position; green: matching subpatterns of the n-of-m pattern type; black and lowercase letters: arbitrary characters. The activated popup window in the figure displays the total pattern and four pattern parts of the nucleoplasmin NLS pattern [Prosite:PS00015].

3of5 supports the input of single or multiple sequences in FASTA formats, alternatively of a single sequence as simple text without header. Patterns can be written in three formats: (1) In a "text only" format each line is interpreted as a distinct pattern. (2) A greater number of patterns can be included in the "FASTA" format with a header line. Then the output of the matches is arranged in the order of sequences, the sequence positions of matches, and by the patterns in their order of input. The sequence is provided for every match. Individual parts of the solutions are marked in color code to discriminate between the distinct parts of the patterns. (3) As third formatting option, individual patterns can be grouped ("FASTA grouped") with the symbol ">>" that serves as grouping element (Figure 4). Several groups can be created within one query. The output of matches is then given for each grouped pattern individually (Figure 5). Pattern descriptions can also be viewed in separate windows (via Javascript) of the result page, which help especially in cases of longer result lists.

3of5 contains three new features in peptide pattern matching. These are: (1) the new peptide pattern type n-of-m, (2) the ability to find all possible solutions for length-ambiguous peptide patterns, and (3) the option to group patterns with similar features in input and output.

### The new pattern type n-of-m

Limitations of software and programs frequently determine the comprehensiveness of the questions that can be applied in the analysis and consequently the completeness of detected solutions. In pattern matching such limitations are to a great extent caused by the inability to exactly describe all variations of pattern ambiguities in regular expressions. More complicated patterns are thus frequently described as mere text supplements within databases and can not be applied in searching. In consequence many protein patterns may have gone unnoticed since no tools had been available to facilitate their detection.

The implementation of n-of-m was originally based on the description of the nuclear localization sequence (NLS) of nucleoplasmin. The commonly employed definition [Prosite:PS00015] of the nucleoplasmin NLS describes two basic residues, a ten residue spacer and a second basic region that contains at least three basic residues in a stretch of five ("3 of 5") positions [23]. This definition contains a number of ambiguities that are due to the variable composition and positions of basic and non-basic residues within the stretch of five residues. Eighty different unambiguous RegEx patterns were needed to cover all possible solution, and there would be still ten different expressions necessary to describe this pattern with common ambiguous RegEx terms. Therefore, the comprehensive definition of such patterns that contain variable arrangements of specific elements is a general problem, when these elements vary in their position, their order and in their content within a stretch of defined length.

The 3of5 application for the first time allows for a complete description of such patterns in one expression, using the n-of-m pattern type. The standard syntax "(nofm)(ABCD)" comprises two pairs of brackets. The first pair contains information on the length m of the pattern and on the minimum number of occurrences n for those characters, which are defined between the second pair of brackets. The content of the remaining, unspecified positions is arbitrary. The complete nucleoplasmin NLS could consequently be expressed as " [KR] [KR].{10}(3of5)(KR)". While for instance Psort II covers this pattern with a predefined expression, this or other programs do not permit for all necessary variability or to search for other patterns of the type (nofm)(ABCD) at all. For example, the pentapeptide pattern "(3of5)(KR)" can also occur in another biological context as part of a mitochondrial localization sequence [24] but is not defined in Psort II.

With 3of5, now any pattern can be comprehensively described, where a defined number of specified residues occurs within a sequence segment by modifying the numbers and characters of the n-of-m pattern type "(nofm)(ABCD)". This includes motifs, as series of amino acids with a typical biochemical character in a given stretch, like charged residues. Thus it is possible to search, for instance, for an octapeptide stretch that contains four basic amino acids. The n-of-m pattern type can be combined with other regular expressions to further expand the spectrum of possible search patterns. This shall be demonstrated again with the nucleoplasmin NLS pattern. Dingwall reported the length of the spacer region between the two basic compounds not to be mandatory 10 residues [23]. Its size can rather range from 9 up to 37 amino acids depending on the respective gene and species. Prosite merely tolerates spacer lengths in the range between 8 and 12 positions in its search for the nucleoplasmin pattern. In contrast, 3of5 permits to freely define the spacer length i.e. ".{9,37}" for this pattern, depending on the respective biological question. Furthermore, the identification of NLS patterns with rotated basic compounds around the linker region is possible [25].

Further pattern definitions can be easily added to enhance pattern specificity. The following examples may demonstrate the effects.

1. The protein tyrosine kinase phosphorylation site exists in the documentation entries of Prosite in the two variants " [RK].{2} [DE].{3}Y" and " [RK].{3} [DE].{2}Y" [Prosite:PDOC00007]. However, the use of these two patterns in the actual search algorithm of Prosite is generalized with the following more simple description " [RK].{2,3} [DE].{2,3}Y" [Prosite:PS00007]. In consequence, the hits obtained with this term consequently contain a number of matches comprising either two or three characters in both of the two variable positions. We obtained 9,640 matches in 5,062 sequences applying this Prosite term to search for tyrosine kinase phosphorylation sites in the human sequence subset of Swiss-Prot (Release 46.1) [26]. Then we repeated the search with a n-of-m pattern which is formulated as " [RK].{2}(1of2)(DE).{2}Y" and which covers the two relevant variants and explicitly excludes the false positive solutions that contain either two or three characters in both of the variable position. We obtained 4,464 matches in 3,253 sequences. This discrepancy can by attributed to the higher specificity of 3of5 compare to Prosite, as only solutions having content-ambiguous spacers of two residues in the first and three in the second length variable position are allowed, or vice versa. We conclude that about half of the matches identified by common syntax of Prosite were false hits that had originated from the limited stringency of the pattern definition in Prosite.

2. The glycosaminoglycan attachment site pattern [Prosite:PDOC00002] is defined in Prosite with the expression "SG.G". Manual annotation in the Prosite database contains additional information; two acidic residues are required at positions -2 to -4, relative to the serine. This information is not implemented in the search tool. Thus, the complete pattern can not be searched for with the Prosite definition, but it can be fully described now as an n-of-m pattern with the expression "(2of3)(DE).SG.G". The Prosite search for PDOC00002 in the human sequences of Swiss-Prot had 3,758 matches in 2,490 sequences. Only 112 matches in 108 sequences were obtained when the same dataset was searched with the 3of5 application. The number of relevant matches in the Prosite search is thus less than 3%.

While patterns with only a small number of variable positions could be expressed also as a number of individual regular expressions (i.e. three for the glycosaminoglycan attachment site, ten for the nucleoplasmin NLS), these numbers would become unmanageable for patterns that contain a greater number of n-of-m-like ambiguities.

The syntax of the n-of-m pattern type has been further extended. This extended syntax of the n-of-m pattern type permits the definition of a pattern part with different numerical constraints that apply to different characters or groups of characters.

When combined with the excluded subset of characters feature it is now possible to describe any pattern in an highly sophisticated manner. The extended syntax may be expressed for instance as (nofm)((operator p)(ABCD)(operator q)(EFGH)) [^J] for a pattern example of the length m, which should include two different groups of characters, each with four characters allowed and constrained by the operators p and q. No characters of the succeeding excluded subset of characters, here "I" and "J", are tolerated in any position. For every character or group of characters the original arrangement of the standard syntax is maintained using two pairs of brackets: The first pair contains information on the number of occurrences for the respective characters, which are defined between the second pair of brackets. This number of occurrences can be constricted by the operators "min" (meaning "minimal" = "equal or more "), "max" ("maximal" = "less or equal") or "eq" ("exactly equal"), followed by the respective limit values (p, q). More than one of these double pair of brackets may be arranged successively. This list of brackets has to be framed by a main pair of brackets. In addition a preceding pair of brackets defines the total length m of the pattern stretch in the form (nofm). Here the length number m is the only true variable parameter in this bracket while the non-variable term "nof" functions simply as a connection to the standard syntax.

The standard syntax of n-of-m is sufficient to define patterns for instance of the nucleoplasmin type as well as of the SV40 large T antigen pattern "pat7". The extended syntax enables to express also patterns like "pat4" of the SV40 large T antigen pattern [21], a pattern composed of 4 basic amino acids ("K" or "R"), or composed of three basic amino acids and either "H" or "P" by the pattern. The respective n-of-m-syntax is to comprehensively describe this pattern is (nof4)((eq3)(KR) (eq1)(KRHP)).

While 3of5 allows for the definition of highly variable sequence patterns it should not be mixed up with so-called "fuzzy patterns" that simply allow for the substitution of letters at individual positions by scoring systems.

### Increased fidelity for peptide patterns with length ambiguities

**Figure 6:**
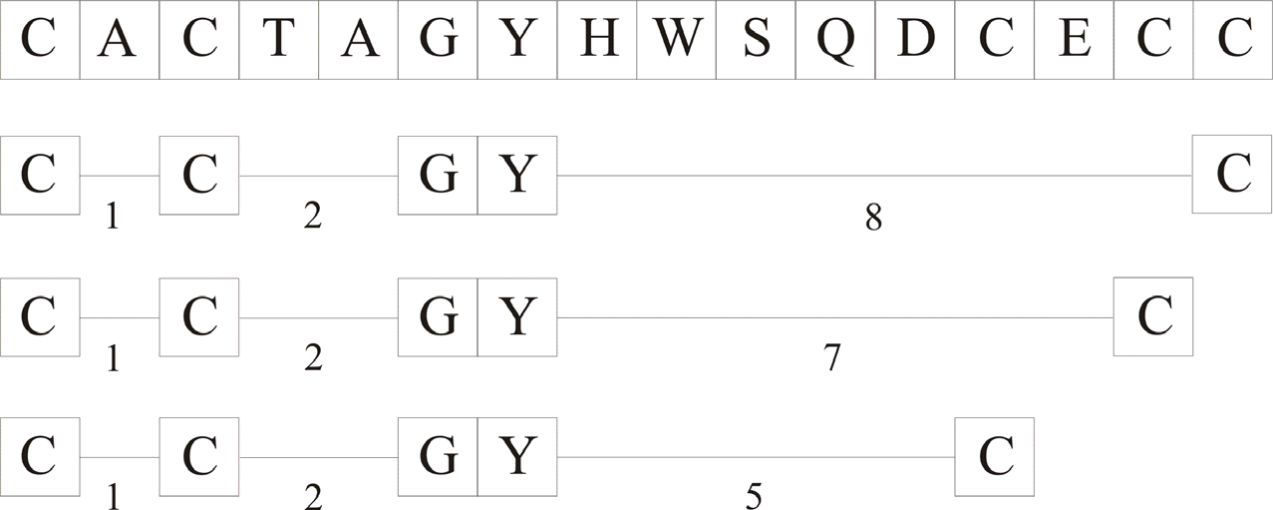
**A length-ambiguous pattern and the derived solution cohort. **The length ambiguity ".{4,8}" within the EGF-like domain signature 2 [Prosite:PS01186] "C.C.{2} [GP] [FYW].{4,8}C" may lead to more than one match per sequence position. For example, the sequence of the tumor necrosis factor receptor [Swiss-Prot:Q9Y6Q6] has three solutions (a-c) which thus form a solution cohort. The sequence parts of arbitrary content are displayed as numbers in the solutions.

Several solutions sharing the same start position in the query sequence are possible in searches when the peptide patterns include length ambiguities. We call a complete set of solutions from such pattern matching a solution cohort (Figure 6). Common regular expressions are often not able to find all solutions. Due to the default settings the RegEx engine only finds the longest solution. This default can be inverted adding the operator "?", then reporting the shortest solution. RegEx engines consequently require two distinct regular expression terms to find the two extreme solutions, while any solution of intermediate length will always remain undetected. However, the more length ambiguities are defined in the pattern and the larger their defined variability in length is, the higher can be the number of solutions in the solution cohort. Prosite at least considers the two extreme possibilities by providing the choice between the two search modes described above. However, there is currently no easy-to-use web-based application for protein sequences that would find further solutions of intermediate length. A solution cohort was the result of relatively short length ambiguities within the pattern in the example shown in Figure 3. The probability for the occurrence and relevance of such solution cohorts however increases with enlarged numbers of length ambiguities and with growing complexity of the pattern. This is especially true for composite peptide patterns that consist of a combination of several individual patterns occurring in variable distances.

3of5 also allows to group peptide patterns using ">>" as grouping element on top of the AND-linkage, where all patterns need to be present to make a match. This creates an OR-linkage. In consequence, user-defined combinations of patterns or groups of patterns are searched for, and the output is ordered in these groups. The grouping of results is beneficial especially in case of long lists of patterns or solutions.

### Comparison with other RegEx-like applications

The Prosite application has been become the gold standard in the field of peptide pattern matching. However, Prosite is not capable of dealing with the n-of-m pattern type. It can only perform pattern matching for patterns that are implemented without leaving an option of modification.

There are currently further tools that perform peptide pattern matching in a sophisticated, RegEx-like manner. However, none of these covers all the features of 3of5. In particular, the combination of rigid rules and flexibility offered by the n-of-m pattern type is not implemented in any other application of peptide pattern matching. For instance, PatMatch provides common features for peptide patterns as subsets, multiplicators and exclusions. However, n-of-m pattern features within larger patterns can not be defined. While a mismatch option is available, such mismatches are always allowed to occur at any position of the total pattern, and cannot be restricted to subpatterns like n-of-m. The extended features of n-of-m can not be addressed with PatMatch either, and in case of length-ambiguous patterns only the shortest solution will be shown. PepPat is an application which integrates common RegEx-like patterns but also this program cannot construct any n-of-m pattern type, neither of the standard nor of the extended syntax. The matching is performed only in the greedy mode in case of length-ambiguous patterns. PatSearch currently offers the most sophisticated pattern syntax for nucleotide patterns. However, it does not allow for a content-ambiguity feature to describe subsets of amino acid characters, while IUB ambiguity terms are implemented for nucleotide patterns. The "either/or" operator functions to select subpatterns, but it does not cover content-ambiguities. In consequence there is no possibility to define subsets, neither excluded subset of characters nor n-of-m pattern types. Furthermore, users of PatSearch need to register at the webpage and receive the results by e-mail. In contrast, 3of5 is open and also allows downloading of results in XML.

### Extensions

The modularity of the underlying algorithm of 3of5 (see methods) permits to develop further extensions of the n-of-m pattern type. For instance fixed distances inside of a n-of-m pattern could be formulated separating distinct parts of the n-of-m pattern. This would define numerical constraints over stretches of longer distances with fixed element blocks in between. This and other extensions will be implemented in the future to cope with the growing complexity and comprehensiveness of pattern specifications that shall be applied in searches.

## Conclusion

We introduce the novel pattern type n-of-m with the standard syntax "(nofm)(ABCD)" and the extended syntax "(nofm)((operator p)(ABCD) (operator q)(EFGH))", which can be combined with an excluded subset of characters, and further pattern types using common rules of Perl regular expressions. This allows for the first time to describe ambiguities in a peptide pattern, which arise from alterations in position, order, and content of characters in a pattern stretch of defined length, using only one expression. The n-of-m pattern type results in an enhanced precision in pattern matching, as was shown in comparison with several Prosite patterns applied to the human Swissprot sequence set. n-of-m is implemented as basic part of the web application "3of5" which is generally accessible. This application has an unprecedented fidelity for length-ambiguous peptide patterns. With 3of5 all solutions are found – in contrast to the common pattern matching applications that can merely detect either the longest or the shortest solutions for any starting position in protein sequences. Its easy-to-use web interface makes 3of5 a convenient sequence mining tool towards a refined pattern analysis. The modular structure of the underlying algorithm facilitates extensions that will cover additional n-of-m-like pattern types. Thus the 3of5 application may serve as a module that bridges the gap between empirical experimentation and the theoretical collection of patterns.

## Availability and requirements

- Project name: 3of5

- Project home page: 

- Operating system(s): Platform independent

- Programming language: Perl

- Other requirements: Java scripting

- License: free

- Any restrictions to use by non-academics: no license needed

## Authors' contributions

MS developed the algorithm and the web application, performed the comparison of the 3of5 results with the Swiss-Prot database. MS and SW designed the study and drafted the manuscript. AM and AP helped to draft the manuscript. All authors read and approved the final manuscript.

## Figures and Tables

**Table 1:** Common regular expressions and the n-of-m pattern type in the 3of5 application Individual common RegEx terms are displayed as they can be applied in 3of5. Types of allowed ambiguities in the individual RegEx terms are listed. "no" no ambiguity; "yes" ambiguity can be expressed with that particular term; "any" ambiguity with any residue allowed. Notes: (1) The general term "ambiguity" used in the text is extended here to "content-ambiguity" to distinguish this from the "length ambiguity"

**Different levels of Regex descriptions**	**Verbal description**	**Example of syntax**	**Potentially content-ambiguous^(1)^**	**Potentially length-ambiguous**
**Description of single positions**	Discrete character in one position	K	no	no
	Subset of characters for one position	[KRH]	yes	no
	Arbitrary character in one position	**.**	any	no

**Description of multiple positions**	Stretch of identical characters, with fixed length	K {3}	no	no
	Stretch composed of a subset of characters, with fixed length	[KRH] {3}	yes	no
	Stretch of identical characters, with variable length	K {1,3}	no	yes
	Stretch composed of a subset of characters, with variable length	[KRH] {1,3}	yes	yes
	Stretch with arbitrary characters, with variable length	**. **{1,3}	any	yes

**Description of multiple positions of n-of-m**	Stretch composed of a subset of characters that need to be present with a defined number of matches within sequence of otherwise arbitrary composition, with fixed length	(3of5) (KRH)	yes	no

**Description of multiple positions of n-of-m in extended syntax**	Stretch composed of different subsets of characters that need to be present with defined numbers of matches within sequence of otherwise arbitrary composition, with fixed length	(nof5) ((min3) (KRH) (max1) (P))	yes	no

**Restriction of content of single/multiple positions**	Any stretch describable by a pattern which should not contain the characters defined in the [^ ] brackets	[AGC] {2,5} [^KRH]	no	no

**Restriction of position of total pattern**	Pattern begins at sequence start	^ KKK	no	no
	Pattern ends at sequence end	KKK $	no	no
